# Evaluating the impact of the supporting the advancement of research skills (STARS) programme on research knowledge, engagement and capacity-building in a health and social care organisation in England

**DOI:** 10.1186/s12909-024-05059-0

**Published:** 2024-02-08

**Authors:** Gulshan Tajuria, David Dobel-Ober, Eleanor Bradley, Claire Charnley, Ruth Lambley-Burke, Christian Mallen, Kate Honeyford, Tom Kingstone

**Affiliations:** 1 Research and Innovation Department, Midlands Partnership University NHS Foundation Trust, St George’s Hospital, Block 7, Corporation Street, Stafford, ST16 3AG UK; 2 School of Medicine, Keele University, David Weatherall Building, Newcastle, ST5 5BG UK; 3 College of Health and Science, University of Worcester, Henwick Road, Worcester, Worcestershire, WR2 6AJ UK

**Keywords:** Research capacity development (RCD), Continuing Professional Development (CPD), Research skills development, Barriers to research engagement, Evaluation, Evidence-based practice

## Abstract

**Objectives:**

To evaluate the impact a novel education programme - to improve research engagement, awareness, understanding and confidence - had on a diverse health and social care workforce. Barriers and facilitators to engagement were explored together with research capacity-building opportunities and ways to embed a research culture. The programme is entitled ‘Supporting The Advancement of Research Skills’ (STARS programme); the paper reports findings from a health and social care setting in England, UK.

**Methods:**

A four-level outcome framework guided the approach to evaluation and was further informed by key principles of research capacity development and relevant theory. Quantitative data were collected from learners before and after engagement; these were analysed descriptively. Semi-structured online interviews were conducted with learners and analysed thematically. A purposive sample was achieved to include a diversity in age, gender, health and social care profession, and level of attendance (regular attendees, moderate attendees and non-attenders).

**Results:**

The evaluation spanned 18 half-day workshops and 11 seminars delivered by expert educators. 165 (2% of total staff at Midlands Partnership University NHS Foundation Trust (MPFT)) staffs booked one or more education sessions; 128 (77%) including Allied Health Professionals (AHPs), psychologists, nursing and midwifery, and social workers attended one or more session. Key themes of engagement with teaching sessions, relevance and impact of training and promoting a research active environment were identified with relevant sub-themes. Positive impacts of training were described in terms of research confidence, intentions, career planning and application of research skills as a direct result of training. Lack of dedicated time for research engagement, work pressures and time commitments required for the programme were key barriers. Facilitators that facilitated engagement are also described.

**Conclusions:**

Findings demonstrate the impact that a free, virtual and high-quality research education programme had at individual and organisational levels. The programme is the product of a successful collaboration between health and social care and academic organisations; this provides a useful framework for others to adapt and adopt. Key barriers to attendance and engagement spoke to system-wide challenges that an education programme could not address in the short-term. Potential solutions are discussed in relation to protecting staff time, achieving management buy-in, recognising research champions, and having a clear communication strategy.

**Supplementary Information:**

The online version contains supplementary material available at 10.1186/s12909-024-05059-0.

## Background

Research has played a pivotal role in the advancement of health and social care by, for example, informing early diagnosis, the development and testing of new treatments for prevention, cure, recovery and palliative care [1]. The importance of research is heralded by key health and social care bodies in the UK, the context for this paper. The UK Government policy paper on clinical research delivery identifies the need to: ‘support healthcare professionals to develop research skills relevant to their clinical role and to design studies in ways which ensure delivering research is a rewarding experience, rather than an additional burden’ [2]. The Chief Nursing Officer for England’s strategic plan for research also emphasises the importance of developing a culture where research is relevant to all nurses, either through direct involvement or the use of research evidence as a key element in professional decision-making [3]. Similarly, the Royal College of Physicians [4] states that healthcare providers should see research as an integral element in care delivery, and to emphasise its ongoing commitment to social care research, the NIHR became the ‘National Institute for Health and Care Research’ in April 2022. The response from the research community to the Covid-19 pandemic has further boosted the impetus and appetite for health and social care to embed global and multi-disciplinary research strategies for the future [5].

Having sufficient research capacity and capability is important to enabling health and social care services and workers to translate research into practice [6]. However, inequalities exist in so far as research is not perceived as accessible and inclusive by all. Several studies describe workplace barriers including time [4, 7, 8] resources, such as access to published research [8, 9] and lack of research knowledge, experience and expertise, both in terms of carrying out their own research and putting the findings of published research into practice [9]. Some professional groups describe lack of access to relevant training as a barrier to developing research knowledge and skills, (e.g. nurses [8–10]). Fry and Attawet [8] also identified a lack of organisational and management support for research linked to the absence of a culture that promotes research as an integral part of clinical practice. Thus, to nurture research engagement an individual (bottom-up) and service-level (top-down) approach to research capacity development (RCD) is necessary [11].

A recent evaluation of National Institute for Health and Care Research (NIHR) funding awards suggested that whilst funding could be transformative and contribute to a healthy research culture in health and care organisations, issues of inequality were identified by professionals working in specialisms with less research experience or expertise. These were in organisations without connections to more research-intensive universities and by those working in non-medical professional groups (e.g. Allied Health Professionals (AHPs), nurses) [12]. This was further highlighted by a study with social care staff, which found they valued research but showed low levels of engagement and skill [13]. Authors would like to highlight here that they recognise that social care staff and social workers provide different functions. Social workers aim, “to provide support for people to help them to deal with the personal and social issues which affect their lives”… whereas “Social care is one of the terms which is used to refer to the strategies which are used to help to care for people who are in need” [14]. Even though these terms may be used sometimes interchangeably they are different in terms of qualification required to attain the title and the duties they perform. A growing evidence base identifies the key mechanisms to support Research Capacity Development (RCD) in health and social care. A rapid evidence review [15] highlighted intrinsic factors (e.g. attitudes and beliefs) and extrinsic factors (such as recognition of research skills acquisition within career progression and professional development via professional bodies, creation of personal awards); and observation of impacts on practice as helpful to encourage NHS staff to engage with researcher development.

### Context to the STARS programme

**Fig. 1:**
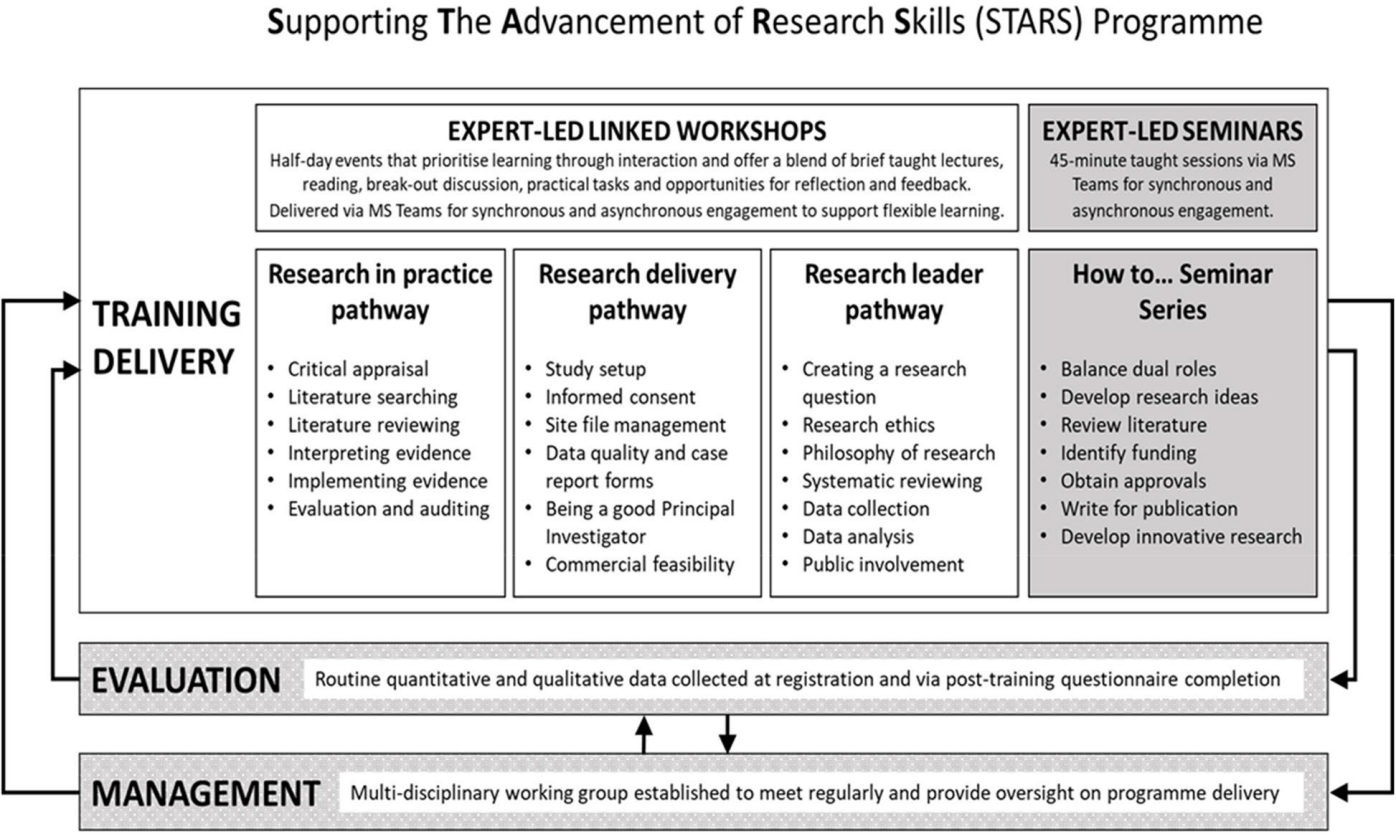
Supporting the advancement of research skills (STARS) programme This paper reports findings from the evaluation which aimed to evaluate the delivery of the STARS programme to assess delivery outcomes, understand learner experiences, facilitators and barriers to engagement, and future opportunities

MPFT is a health and social care NHS trust with a track record in research delivery and is in the process of developing research leadership. At the time of writing, the NHS Trust had not achieved university hospital status, although it works closely with two universities which developed the STARS programme in partnership (see Fig. 1 and Supplementary File 1 for a full overview of the structure of the programme). The STARS programme provides a useful resource to address disparities in research engagement between different professional groups in health and social care. Despite more opportunities for research having been generated for nurses and AHPs by organizations such as the NIHR Collaborations for Leadership in Applied Health Research and Care (CLAHRCs) and Clinical Research Networks (CRNs), disparities persist between non-academic clinicians and the opportunities available to certain clinical specialities [16–18]. Challenges and barriers to research training engagement highlighted in this paper are likely to have global relevance [19]. Thus, more broadly, offering programmes such as STARS may also help address global disparities in research engagement given the UK has the highest percentage of doctors (28.6%) and nurses (15%) who are trained in foreign countries [20]. STARS was designed in consultation with staff to identify existing barriers to engagement in research training, provide all staff with improved access to high-quality research training to enhance their confidence in research and enable the best use of empirical evidence in practice. The STARS programme was launched in January 2021.

## Methods

### Design

The approach to evaluation of this training programme was informed by Kirkpatrick’s four-level outcome framework: reaction (was training enjoyed?), learning (did learning occur?), behaviour (did behaviour change?) and results (was performance effected?) [21]. As this is a new programme, data was gathered against the first three levels of Kirkpatrick’s evaluation model. Contemporary criticisms and revisions of the model were incorporated to better understand the chains of evidence and wider contextual factors that may influence the delivery of a new programme [22], such as the STARS programme.

### Data collection

#### Quantitative data

Data including information such as highest educational qualification, job role, the reason for attending and the line manager’s approval to attend the training was collected at the point learners registered for a teaching session. Data indicating service areas, rate of dropouts, staff backgrounds, highest and lowest rate of attendance was collected from the attendance record. Data was also collected using a brief post-session feedback (see Supplementary material- Learner Evaluation Form) form, which included a likert scale question inviting learners to rate the quality of the training.

#### Statistical analysis

Quantitative analysis was performed at a descriptive level, using Microsoft Excel (2016).

#### Qualitative methods

Semi-structured interviews were conducted with programme participants to explore learner experiences (aligned with Kirkpatrick’s reaction level), outcomes (learning) and intentions to apply research knowledge (*intended* behaviours). Flexible interview formats were offered to encourage participation, such as online interviews and providing responses via email. Interviews were facilitated using a topic guide (see Supplementary material- STARS Interview Guide) that was iteratively revised.

#### Recruitment and sampling

A purposive sample of participants was identified using data from the programme booking form and attendance records:

- Regular attenders: Those who attended a minimum of five teaching sessions across the whole programme or a single pathway.
- Occasional attenders: Those who attended very few (1–2) sessions across the whole programme.
- Non-attenders: Those who registered to attend, but eventually didn’t attend, to explore barriers to engagement.

Participants were invited by email for a maximum 30-minute interview. All potential participants were emailed a participant information sheet. They were given time to read the information and a contact name for any questions related to their participation, before being asked to confirm their participation in the study.

#### Description of sample

Thirty-six staff members were categorised as *regular* attenders; all were invited to take part in an interview. Two individuals declined to participate citing a lack of relevance, as they left their learning events halfway; two individuals declined due to work pressure following illness; three were ‘out of office’ according to email replies, and no response was received from 14 individuals. The remaining 15 agreed to participate in an interview with 10 choosing to use Microsoft Teams and five to provide written responses- ‘email interviews’. This method is becoming increasingly used to help supplement other forms of data and support involvement of healthcare professionals, who may have limited time/capacity for research but valuable knowledge to share [23, 24]. Participants represented a diverse range of professional backgrounds, including: AHPs, psychologists, nursing and midwifery, and social workers; this reflected the broad range of learners on the programme. A semi-structured interview guide was used. The interviews were audio recorded and later transcribed in full by the lead author (GT).

*Occasional* (*n* = 17) and *non-attenders* (*n* = 13) were invited to participate in an interview. These were staff members who had booked several teaching sessions (1–12) but either did not attend any with/without apologies (*n* = 30) or attended only one or two. Seven email addresses were not valid as the staff had either left the service or changed role; four had an automated ‘out of office’ response set; four staff declined to participate and there was no response from 11 email addresses. Five staff agreed to be interviewed. A brief topic guide was used with questions aiming to find out just the reason/s behind non-attendance in the training. As these interviews were brief, non-verbatim notes were taken by the interviewer and included in analysis. At the end of each of these interviews, the notes were validated with the interviewee.

#### Qualitative analysis

The data analysis followed a thematic approach [22] to identify key themes. Data-driven coding was conducted to establish meaning from the words of participants; coding was also informed, a-priori, by the levels of the evaluation framework [25]. Initial coding was done by GT and TK who read all transcripts to support familiarisation before generating an initial set of codes. Right from initial codes to final themes, other than the authors, the wider STARS team gave input in various Team meetings. Similar codes were then compared and grouped to identify initial themes; these were reviewed to shape a preliminary set of main themes. Preliminary themes were shared and discussed with the team before finalising.

## Results

### Quantitative findings

#### Attendance

Over the 12-month evaluation period, a total of 18 half-day workshops were delivered, six from the research in clinical practice pathway; four from the research delivery pathway; eight from the research leader pathway (refer to Fig. 1); and 11 seminars to support the development of key skills. In total, 165 (2% of total staff at MPFT) staff members booked one or more teaching session. 128 (77%) attended one or more teaching session. On average, sessions in the research in practice pathway were attended by 25 staff; 12 in research delivery pathway; 21 in the research leader pathway; and 17 in seminars.

#### Qualifications, backgrounds and expectations

According to the booking form, attenders represented a range of professional groups.

- Nursing registered − 29 (23%).
- AHPs − 23 (17%).
- Additional clinical services (all healthcare services) − 21 (16%).
- Additional professional scientific and technical (such as pharmacist, qualified psychological therapist, social worker etc.) -15 (12%).
- Medical profession − 14 (11%).
- Other (e.g. research staff) − 26 (20%).

Approximately 85% of staff had reported prior educational qualifications, the majority included: 20% (*n* = 33) bachelor’s, 19% (*n* = 31) master’s, 3% (*n* = 5) doctoral degrees, 6% (*n* = 10) diplomas and nearly 2% (*n* = 3) MBBS (Bachelor of Medicine, Bachelor of Surgery); remaining attenders did not provide information on their educational background.

### Explanations for booking the training and number of staff

At the time of booking the course, staff were asked to provide reasons and expectations from STARS sessions using an open text box. Descriptive analysis of responses is presented in Table 1:

**Table 1:** Reasons for booking the training and number of staff

Reasons for attending STARS	Number of staff *n* = 151
Help better understand/access research for developing practice	32
Additional support for academic work (e.g. critiquing a paper; completing systematic reviews)	25
Support for developing research in Trust	22
Career progression	18
CPD (Continuing Professional Development)	19
Personal interest and development	29
Partnership/commercialisation element of the STARS programme to be used in designing e-learning within trust	4
Line manager’s recommendation	2

A better understanding of research in practice, additional support for academic work and the development of research in trust were the most common reasons provided (Table 1).

### Post session evaluation feedback

Learners demonstrated their learning from the sessions in a variety of ways and more often using the session specific feedback. In total, 195 feedback forms were completed and covered 24 sessions. The number of ratings completed per session ranged from 1 to 25. 136 (70%) learners rated the session they attended as ‘very good’, 52 (27%) rated as ‘good’, 4 (2%) rated ‘adequate’ and 2 (1%) rated ‘poor’. Qualitative findings, presented below, help us to make sense of the session ratings.

### Qualitative findings

The main themes and sub-themes from the analysis of qualitative data from interviews are summarised in Table 2 and described with illustrative quotes in the following sections.

**Table 2:** Main themes and sub themes

1. Engagement with teaching sessions	• Factors considered while selecting the sessions • Barriers to attendance and engagement • Facilitators to engagement
2. Relevance and impact of training	• Training content, relevance and suitability • Impact on knowledge, skills and attributes • Applying new learning
3. Promoting a research-active environment	• Research career pathways • Workforce satisfaction • Improving Awareness about research support services

### Engagement with teaching sessions

The reasons given by staff attending the training in booking forms (Table 1) and discussed in interviews were reflected to a large extent in the way participants chose the teaching sessions they attended. Eight interviewees had received research training as part of their degree-level qualifications; one was currently involved in conducting research at work.

#### Factors considered while selecting teaching sessions

Some staff were much focused on what they wanted to take from teaching sessions and booked selectively; however, some wanted to attend all, indiscriminately, due to unequal access in such training opportunities in the past and/or in their departments:

*“I wanted to do them all because my concern is that they might not be offered again because we’ve never had them in social care… we’ve never had researchers come and talk to us in social care and social work unless you go to Uni.”* P 4.

Some staff described their learning as focused on intrinsic factors such as:

*“It’s always good to update because I think you find your own way in doing things like informed consent.* P 11.

For other staff, learning on the programme was driven by extrinsic factors like:

*“Social work and social care does have a huge gap in terms of research participation. We are trying to develop that within the organization and regionally”* P 13.

Relevance of a teaching session to the current role was considered before booking by staff who either had knowledge or were currently involved in doing research but the staff without previous opportunities like this booked relatively indiscriminately. Intrinsic factors such as personal interest and career progressions and extrinsic factors such as organisational development were additional reasons to attend the teaching sessions.

#### Barriers to attendance

Getting data from those who did not attend after booking proved difficult. Four staff declined to take part in evaluation interviews because of work pressure or illnesses; this may reflect some of the reasons for non-attendance. Another five agreed to take part in short interviews to discuss their lack of attendance with the programme. All interviewees pointed towards time pressure as the main issue.

Qualitative data from the interviews with the regular attenders about barriers to attending some of the training after booking revealed similarities in reasons as the non-attenders. A general lack of time due to staff shortages highlighted the role of the line manager’s approval in attending the training as discussed by two staff members:

*“some sessions that I could not attend as my manager didn’t think I should attend so many sessions, because of the pressures of the service following the covid backlogs etc”* P 5.

One staff member briefly raised the issues of empowerment where some staff might find it difficult to get the line manager’s approval to attend such training:

*“And perhaps you need to get the buy in from the managers, because there’s an awful, awful lot of staff that aren’t really empowered to be able to go off and do this and then influence their work”* P 7.

Communication and marketing of the new training was highlighted as a barrier to attendance by staff from one of the departments:

*“I think one was probably in the promotion, I came across it by chance…that’s something to do with our organization because it kind of sits slightly outside of MPFT, so I think sometimes that messaging doesn’t always get through” P3*.

Prioritising paid training over STARS training was also a reason for one of the staff to miss some of the teaching sessions:

*“I’ve missed some STARS trainings because of attending other trainings which are paid training or conferences that have cost money. So obviously I’ve prioritized them over some of the STARS training”* P 9.

#### Barriers to engagement

Providing training across different professional groups highlighted difficulties in understanding respective languages. Two respondents reported that some content used clinical language that was difficult to understand:

*“There’s also an element of understanding research and how it can be applied there’s probably an element of language as well, so it’s not just clinical…or health orientated, it’s also care. So it is just understanding that language barrier so that social work and social care staff know that it’s appropriate for everybody in the organization”* P 13.

For one staff member the pace of delivering the graphic and statistical information teaching was very fast and difficult to understand:

*“Sometimes it felt like the presenters for some statistical information went too fast when that was the area that most people are weaker on, so perhaps some courses tried to fit too much into one session”* P 5.

A couple of staff discussed the workshops as disengaging due to long presentations and less interaction:

*“the ones where you will just kind of like listening for three hours. They were really hard to stay engaged with”* P 9.

For two staff the breakout rooms were not as helpful as explained by one:

*“it can be awkward when you’re with people you don’t know and haven’t got a full grasp of the subject, and trying to think of contributions”* P 5.

One staff also highlighted how attending the training from a shared office space can be problematic compared to a private space:

*“As when doing it in the office, it’s harder to engage in group discussions due to fear of disrupting other colleagues”* P 2.

Other ways of delivering the training were also suggested due to long commitments for the workshops. Two participants suggested that three-hour workshops were too long when delivered online; face-to-face learning was preferred:

*“it would be nice to have it when we can to have some classroom based stuff because again, it just feels more natural to ask questions and you get to have those conversations in breaks”* P 1.

And according to one participant, the training could be delivered using pre-recorded content:

*“If there was a way to like the website on the Internet, all these links that you could click on to watch re-watch everything so you know where to go to one place to see all”* P 6.

However, for two participants the recordings of teaching sessions were not as good as attending in real-time, as explained by one:

*“You’re not the one engaging in it like because obviously you’re just watching it after the fact, so I don’t sit through the whole thing…If you’ve got questions, there’s nowhere to ask those questions”* P 9.

#### Facilitators to engagement

Online synchronous delivery of the teaching sessions was valued by all interview participants, in the context of the Covid-19 pandemic. Use of breakout rooms for small group discussion and interaction was considered useful by most of the interview participants, for example:

*“that was quite nice that you’d catch up with people that you were in the breakout rooms and could get to know a bit more about what they were doing and so I found that quite helpful from like a networking perspective”* P 10.

Most of the staff members discussed keeping the recorded videos for future reference as very helpful:

*“I know I’m not going to have time to apply myself to do in any sort of research at the moment with how things are at work, but I’ve got all the recordings and so could go back to those”* P 10.

To summarise, barriers to attend the training included a lack of time on the participants’ end and lack of promotion. Perceived value due to no direct cost associated with the training was also revealed as a reason to miss a session after booking. Pace, professional-specific language, length of teaching and shared office space were highlighted as some of the barriers to engagement. Regarding facilitators to attend and maintain engagement, all staff were happy with online delivery and the availability of recordings was useful. However, mixed opinions were shared about the usefulness of breakout rooms given the range of professional groups that the staff belonged to.

### Relevance and impact of training

Staff described various benefits to their research practice since attending STARS sessions, such as, writing and publishing a short report; working on a literature review; signing on to a university course; successfully receiving regulatory research approvals; and completing preliminary work to attend a professional doctorate or equivalent.

#### Training content relevance and suitability

All interview participants commented on the programme content and described it as comprehensive and well-balanced in terms of topics and delivery:

*“I think it was really well balanced. The presenters came from diverse backgrounds and research was treated holistically by all, so everything felt relevant”* P 12.

#### Impact on knowledge, skills and attributes

One participant described how learning was helpful to understand key areas in greater depth:

*“I have an understanding of some critical appraisal and things like that, but it was probably more surface level and the STARS programme helped me to develop that quite significantly”* P 1.

For another staff it helped with attending and presenting at different teaching sessions:

*“So I’ve attended the regional teaching partnership programs we’ve presented our [name] project across [organisation] who are now looking at setting up a regional program. We’ve presented at NIHR events so yeah, definitely useful” P 13*.

The teaching sessions had a prompt impact on the knowledge and skills of those staff who already had some knowledge of research and also those who had identified specific opportunities to put into practice.

#### Applying new learning

Some learning on the training had wider applications that went beyond research, topics such as informed consent:

*“Things like the informed consent training because for all our new staff that’s a major part of research. So from that we’ve drafted kind of a memoirs and processes formally based on sort of training materials on how an informed consent should be conducted so that we know that everybody starting at the same level”* P 11.

Learning on one particular workshop helped to build a participant’s confidence in reading, making sense, and talking about research followed by conducting their own literature review:

*“I used the literature review knowledge that I gained to do a very comprehensive literature review. Very rapid, quite comprehensive and then presented it. So I was able to put it into practice straight away” P 3*.

Overall, most of the participants mentioned using the new learning in practice but only a few staff members were able to provide practical examples.

### Promoting a research-active environment

Staff discussed how they were using more resources from the organisation such as websites, the local research department, and library services in creating a research identity for themselves and contributing towards a research-active environment within and across their respective departments.

#### Research career pathways

The STARS programme helped to awaken ambitions for research and staff showed how keen they were on getting involved in doing research. Participants described doing their own research as a better option when other routes for progression were limited in their department:

*“where I’m at in my role, there isn’t really anywhere to go unless you want to be a team leader, which isn’t really what I want to do. I really enjoy the patient facing side of things, and so I’ve always kind of said I’d be more interested in more specialized role or doing some research”* P 10.

STARS was also useful in the stages of career development and for some it was helpful in starting the new paths as discussed by one:

*“It’s either doing a feasibility or that sort of level today as part of a master’s course or doing their pre doctoral the NIHR sort of work to get a project effectively ready”* P 6.

However, there was also a sense of being unfulfilled among some of the participants:

*“I’d like to progress in it, but it’s where do I take it because I don’t know what opportunities are out there and how to apply for anything really”* P 4.

*“I’m really interested in doing some research in the area that I work in because I feel like there’s lots of improvements and things that could be made with how we do things and for the clients to get the most out of the service…I think with the STARS stuff I’ve sort of parked it so I’ve got it all saved together in a folder like ready so I can go and access it”* P 10.

STARS opened up different routes for career progression for some staff. On the other hand, staff without immediate opportunities to get involved in research reported experiencing frustration because of the fact that there were no obvious opportunities for them to put their improved skills into practice. Success stories (going on a pre-doctoral path; progression for those who were already doing their master’s/doctorate etc.) of those who had some research base highlights the initiation of research identities.

#### Workforce satisfaction

In addition to feeling motivated to complete their academic qualifications, two staff members discussed how much they valued the STARS training and one participant described staying in their job, in order to access the training:

*“I’ve not come across any other type of research training that is like is what the STARS programme offered. I purposely stayed within my role to access this stars training”* P 9.

#### Improving awareness about research support services

The staff interviewees appreciated the associations to other support and resources that they had found out about while attending the STARS training. This included the library services and the R&I team:

*“And the fact that our library helps us is phenomenal…So it’s given me a lot of knowledge about the wide organization and just how invested we are in research and that there are people [R&I] to help”* P 7.

The STARS programme has been developed with contributions from different departments in order to make it suitable for all staff members to access and understand. This was reflected in the discussion where the interviewees appreciated the other links and resources.

## Discussion

The current paper reports findings from a mixed-methods study, which aimed to evaluate the delivery of a novel research training programme to health and social care staff in a single organisation in England (MPFT). The mixed methods approach generated key data against three of Kirkpatrick’s framework (reaction, learning and behaviour). Quantitative findings demonstrated good engagement with the programme from a diverse range of professional groups; a broad range of reasons were given for engagement. All of which demonstrates the broad appeal and initial reaction to the programme offer, particularly among professional groups who may not ordinarily engage in research (e.g. social care, nursing and midwifery staff). Ratings of session quality were very positive with 97% of ratings either very good or good. Qualitative findings highlighted three key themes: engagement with training, relevance, and impact of training, and promoting a research-active environment. Within these themes, positive reactions to training (e.g. appreciation, satisfaction, collaboration with others, access to new resources), evidence of learning (e.g. understanding critical appraisal) and change in behaviour through practical application (e.g. conducting a literature review) and sharing learning (e.g. networking) were identified. However, barriers still exist for many, including research terminology, limited capacity and the need for wider promotional campaigns.

Comparisons with findings from previous research in other areas and with elements of Gee and Cooke [26] framework for Research Capacity Development in health care are made, particularly within the areas of Close to Practice (CTP), Infrastructure (INF) and Skills and Confidence Building, which closely align with our findings and help support transferability to other contexts whilst also realising that a training programme can only do so much.

### Close to practice

Gee and Cooke’s [26] ‘Close to Practice’ principle covers themes such as keeping research relevant to health care and informing day-to-day practice The current programme tried to be inclusive of all professional types (i.e. being close to practice); however, as identified in the engaging with teaching sessions theme, some language barriers were highlighted by staff from social care backgrounds who felt excluded due to the clinical/academic language used to deliver the training session – which may have obscured the relevance of the content for this group of learners. Still, the way the STARS programme supports this principle is evident in the content, which addresses both the main strands of the UK and English health policy, driving increased health and care involved in research:

- the routine use of research findings in day to day practice;
- increased involvement in research activity within the health service.

(referred to by Wakefield et al. [13] as ‘using research’ and ‘doing research’). The findings of the current evaluation demonstrated that participants’ reasons for booking onto the programme usually included one or both elements. Participants’ motivations also mirrored those found by Dimova et al. [15], presenting expectations that the STARS content supported both individual career development and organisational objectives such as high-quality patient care. In line with Ariely et al. [27] and Abramovich and McBride [28] booking but not attending the current training sessions was an indication toward the perceived low value of the training considering it was completely free for the staff. As the training is free to attend for the staff & managers with no direct impact on teams’ budgets, the priority to attend was given to paid trainings over STARS, sometimes.

### Support infrastructure

Gee and Cooke’s [26] ‘Developing a support infrastructure’ principle covers ‘building additional resources and/or processes into the Trust’s organizational system to enable the smooth and effective running of research projects and for research capacity building’. The findings from the current evaluation, particularly under the ‘Promoting a research-active environment’ them, also showed how a wide-ranging in-house research skills training programme open to all staff can help build resources and processes within a healthcare provider that can support greater research activity.

In terms of processes, distinctive features of this training programme were that it was delivered in-house and entirely online. While the move to online training was necessitated by the pandemic (COVID-19), the evaluation showed that online training has the potential to become the delivery method of choice, particularly for in-house training for organisations covering a wide geographical area. Evaluations comparing online synchronous learning to traditional face-to-face learning have generally shown that (though with certain limitations) online approaches can be effective (George et al. [29], found this was the case for post registration medical education). In line with previous research, the current evaluation has also shown that an online-only training programme has challenges but can have a positive impact on applying research skills and developing confidence among healthcare staff [29, 30].

Participants’ feedback identified the importance yet challenge of incorporating interactivity into online training [31–33]. Feedback on the length of the teaching sessions demonstrated that long sessions (in this case two hours or longer) could reduce engagement [33, 34].

The literature on barriers to health and social care staff carrying out either or both of these activities (research finding use or research activity) identified four main barriers:

1. lack of time and/or resources;
2. lack of organisational or management support in other ways;
3. lack of skills, knowledge, and confidence to undertake research or put evidence into practice and.
4. lack of opportunities to develop these skills.

The first two of these are linked to infrastructure, resources and processes. The findings of the STARS evaluation showed mixed evidence in this respect. On one hand, the evaluation echoed previous research [7, 8] that lack of time or staffing pressures was a major barrier to healthcare staff gaining research skills. Lack of protected time for research activities remains an important barrier to embedding a research-active environment into an organisation. As suggested by King et al. [11] the current evaluation was also conducted keeping in mind the long-term impacts on the organisational level. The STARS evaluation found the issue of management support, also identified previously [8], and affected both attendance and opportunities to put skills learnt into practice. On the other hand, the evaluation produced at least one positive example of a manager supporting an attendee in putting skills learnt into practice, resulting in changes in practice.

### Research skills and confidence in the workforce

Gee and Cooke’s [26] ‘skills’ principle covers the provision of training and development opportunities to enable the health and care workforce to develop the skills and confidence to both ‘use’ and ‘do’ research. This principle speaks to the second theme of ‘Relevance and impact of training’ and matches the third and fourth barriers to doing and using research from the research literature mentioned above. This evaluation focused on how the STARS training programme addressed this principle and these barriers.

In terms of the provision of opportunities, the analysis of benefits reported by participants suggest that taking part in the programme contributed to improved skills and confidence in both the ‘using’ and ‘doing’ areas. Comments from the interviews also showed how the STARS programme had addressed the barrier of a lack of opportunities to develop these skills, with two (social care) participants commenting that STARS represented an opportunity not traditionally available to staff from their sector. This helps address one of Wakefield et al’s [13] recommendations about access to research training opportunities.

Previous research [8, 10, 13] showed that a lack of research skills, confidence and opportunities to gain them were issues associated with non-medical staff groups, particularly nurses, AHPs and social workers. However, the opportunity to gain knowledge and new skills through STARS was valued and staff had plans of using them in the future, echoing the results reported by Bullock et al. [35] The analysis of demographic data for the STARS programme was based on broad nationally defined staff categories (United Kingdom Electronic Staff Record (ESR) categories – see ‘A Guide to the Staff Group, Job Role and Area of Work classifications used in ESR’); it was difficult to separate, for example, social workers from other staff categories who usually have higher degrees, a high level of research skills, confidence and knowledge. However, the high level of take-up from nursing and midwifery and AHPs suggest that the STARS programme had been of interest to staff groups that previous research had identified as lacking skills, confidence and training opportunities to make evidence-based practice and research activity part of their working culture.

Comments received in the STARS evaluation raised the dilemma of whether it was possible to make content available and relevant to groups of participants with very different professional backgrounds and levels of research knowledge and experience; or whether attempting to achieve this meant the course content did not meet any group’s needs well. The evaluation found both positives and negatives in this respect – gains from sharing the training with colleagues from very different areas and perspectives versus content failing to suit the needs of the participants, very different prior research and professional knowledge and so inhibiting learning in some cases. Previous research was found, evaluating multidisciplinary training provisions that either spanned a range of professional groups working in the same area or students at a similar stage of education studying in different subject areas [7, 9, 10, 12]. However, no previous research was found evaluating training programmes that matched the STARS participants’ mix of both professional backgrounds and work areas (spanning a range of inpatient and community health and social care settings as well as support services).

### Strengths and limitations

The current evaluation contains both quantitative and qualitative primary data from engagers and non-engagers in a novel research education and training programme for a broad range of health and social care professionals. Qualitative methods were designed to be flexible and pragmatic to capture views from busy health and social practitioners; however, emailed responses did not support in-depth exploration. As the interviewer was also a staff member of the same organisation there might have been some undisclosed responses. Findings report key the components of training that worked/did not work; this information could eventually be used to improve future training in this setting and others. As the participants of the STARS programme and current evaluation are located within a health and social care NHS trust in England, the conclusions are relevant to similar settings only. However, findings seem relevant to non-UK health and social care workers. For example, Withington et al. described how their targeted training and mentoring model enhanced research capacity among social workers [19] Also similar to finding in STARS collaborative approaches have also been discussed as essential by Nystrom et al., in in health and social care context in Sweden to ensure support, trust and understanding among those working in healthcare system [36]. Despite this limitation, the findings highlight how a research training programme can be tailored around the needs of staff and run virtually during a pandemic.

## Conclusions

This evaluation covered a 12-month period in which the STARS programme was rolled out for the first time at MPFT. Findings demonstrate the positive impact that access to free, high-quality, online research education can have in terms of enhancing research awareness and confidence across a diverse range of professional types; some of whom reported unequal access to such training in the past (e.g., social care, nursing and midwifery). Service-level barriers remain that a novel training programme cannot address (e.g., competing burden of clinical roles). It is too early to assess longer-term outcomes relating to the fourth level of Kirkpatrick’s framework (performance) or research culture at an organisation-level; further follow-up research is needed. The STARS programme demonstrates what strong collaboration between NHS and academic institutions can produce and provides a training model that can be adopted and adapted elsewhere to nurture research-active environments and promote research capacity building within and beyond the UK.

### Supplementary Information

**Additional file 1.**

**Additional file 2.**

**Additional file 3.**

## Data Availability

The anonymised quantitative raw data from evaluation registers and qualitative data from interviews is available on reasonable requests. The corresponding and first author, GT, should be contacted if someone wants to request the data from this study.
