# Supplementary material for: Evaluating the impact of the supporting the advancement of research skills (STARS) programme on research knowledge, engagement and capacity-building in a health and social care organisation in England
Source: BMC Med Educ. 2024 Feb 8;24:126. doi: 10.1186/s12909-024-05059-0 (PMC10854097; doi:10.1186/s12909-024-05059-0)
Supplement: Supplementary file 1 — Additional file 1. [file 12909_2024_5059_MOESM1_ESM.pdf]

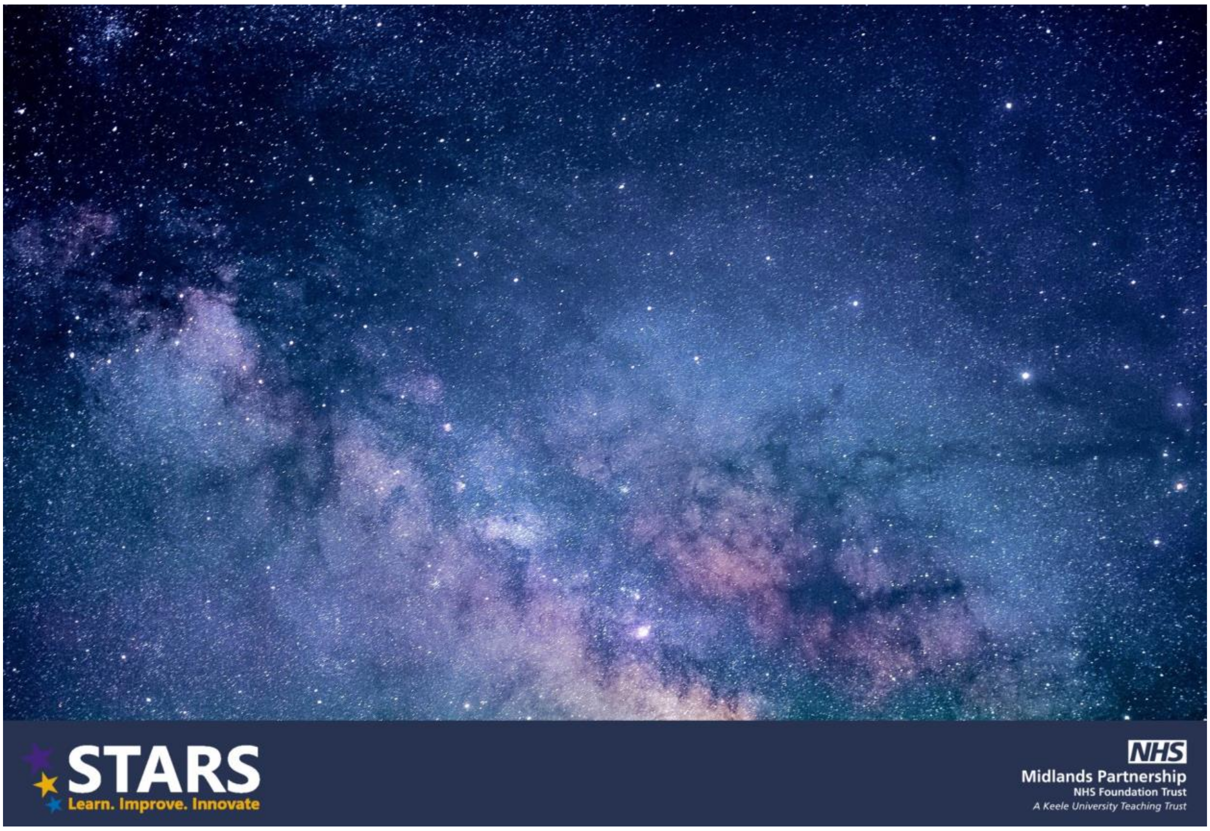

Supporting The Advancement of Research Skills: The STARS Programme Prospectus 2021/22

FOREWORD

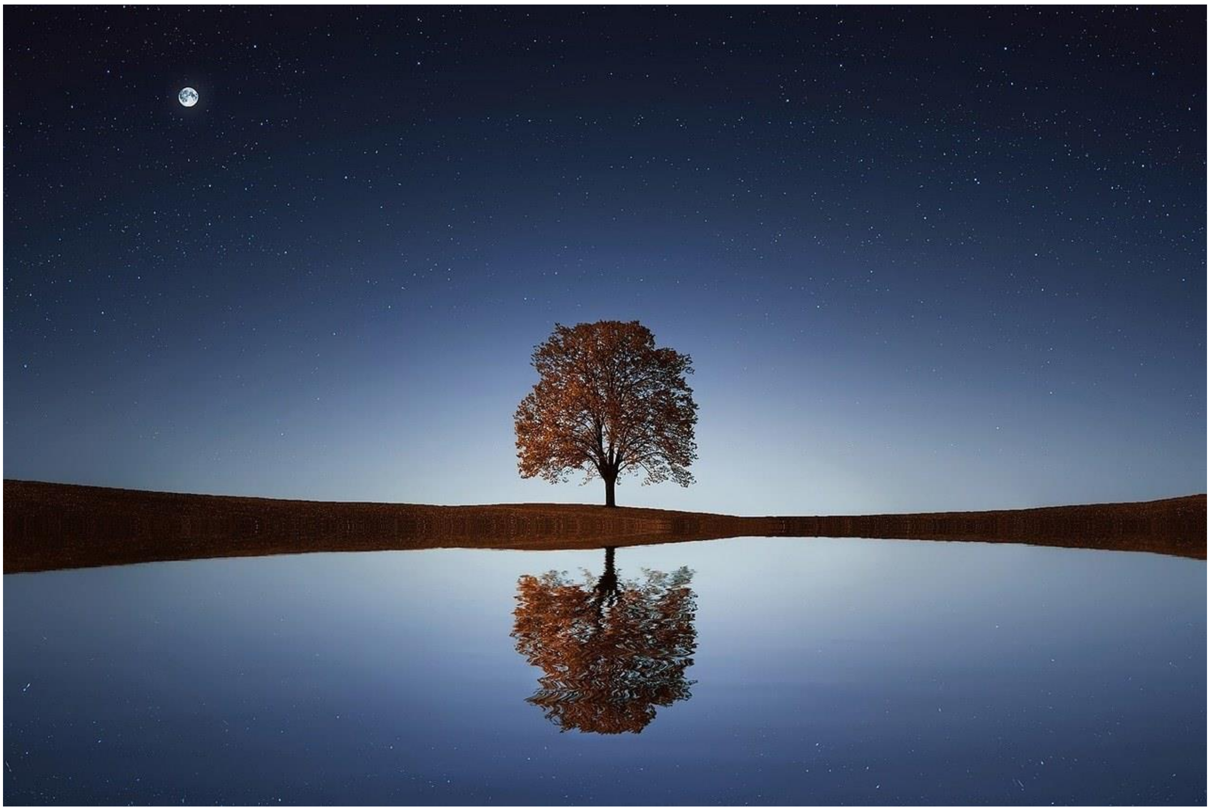

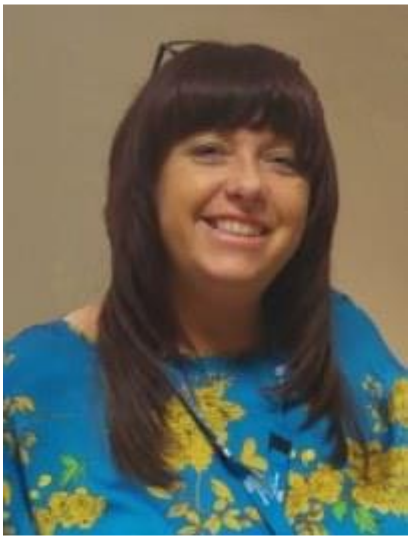

1 - Ruth Lambley-Burke, Head of R&I, MPFT

Dear colleagues,

On behalf of Midlands Partnership NHS Foundation Trust, I am delighted to be able to introduce the prospectus for the STARS programme. The STARS programme represents a fantastic offer to our staff to support their learning and development in research.

The value of research to the NHS and patient care is clear for all to see. We are proud of the active core group of staff already engaged in health research at the Trust. We now want to build on this by promoting wider engagement and by nurturing a dynamic research culture. The STARS programme provides the perfect opportunity to enable this, by offering flexible and accessible ways of enhancing

knowledge, awareness and confidence in research. This is an exciting prospect for staff to obtain new skills and enhance their career satisfaction.

We have carefully considered the needs of staff to ensure there is something in the programme for everyone. ***Please remember! The STARS programme is open to everyone whether you are a clinical or non-clinical staff member.***

We know your time is precious and that our Trust has a large geographical footprint which can make it challenging to attend training. We have taken this and other feedback on board to create a fully flexible programme that enables learners to engage live or via pre-recorded content, whenever and wherever they choose.

We are fortunate to have access to and commitment from a group of leading research specialists willing to share their expertise.

You can find further information about all of these learning opportunities and events in the following pages. If you have any questions, we would love to hear from you via our dedicated email inbox: STARS@mpft.nhs.uk.

Best wishes,

Ruth Lambley-Burke

Head of Research and Innovation, MPFT

"STARS is a comprehensive research training and development programme which will enable staff to learn new skills and develop current ones to support them to build their practice on the best available evidence"

Neil Carr, Chief Executive, MPFT

"The STARS programme demonstrates the commitment of MPFT to ensure that research and innovation are top priorities in the organisation. By providing a new training programme that is accessible to all they are actively investing in a research-active culture that will improve outcomes for patients and support staff development."

Professor Christian Mallen, Academic Director, MPFT and Head of Medical School, Keele University

Welcome to the STARS programme

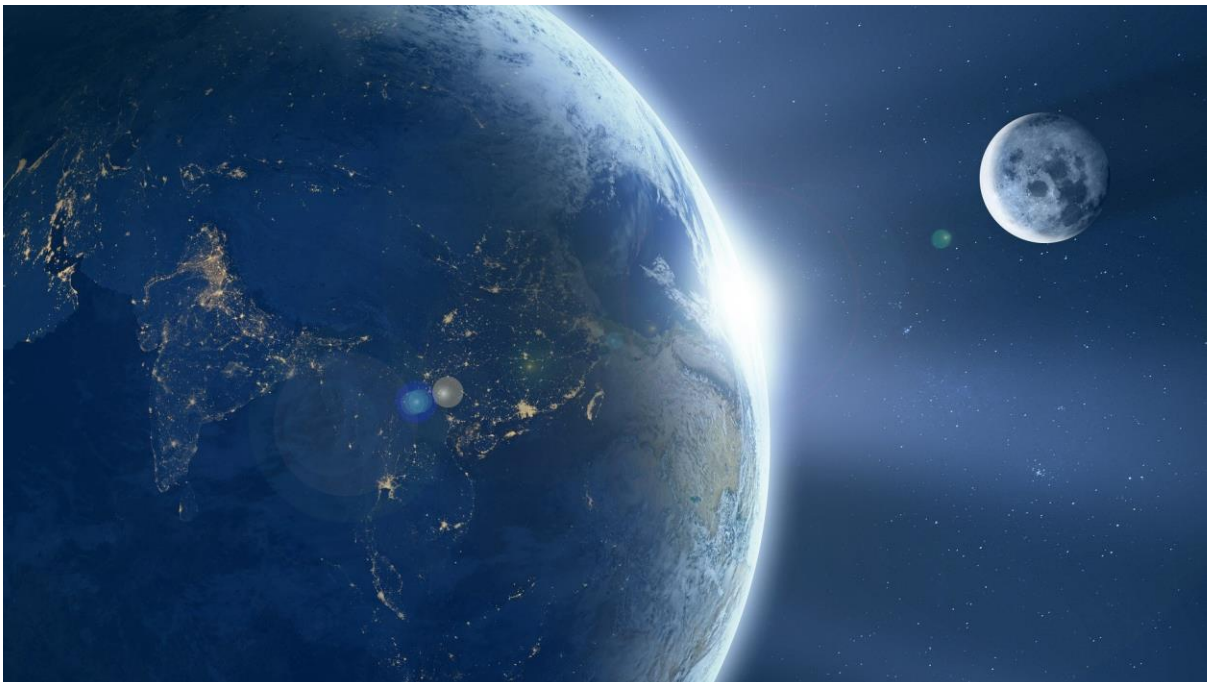

The STARS programme has been developed by the Research and Innovation (R&I) Department supported by academic partners at Keele University and the University of Worcester. The programme has been to designed to achieve the following three primary objectives:

1. *To nurture a positive and engaging research environment at Midlands Partnership NHS Foundation Trust*
2. *To ensure staff have the necessary skills to be able to consider and apply the very latest research evidence into decision making*
3. *To identify and support future leaders in health and social care research*

The programme offers a diverse range of learning and development opportunities and events from structured research methods workshops to clinical-academic knowledge exchange meetings. Learning opportunities will not currently lead to formal qualifications (e.g. postgraduate certificates, diplomas) but can contribute to Continuing Professional Development (CPD).

Three structured workshop pathways have been established to guide staff through tailored learning journeys to either make better use of research in their everyday practice, to deliver existing research projects, or to work towards designing and leading their own research. This prospectus provides a comprehensive overview of these learning pathways and additional learning opportunities that are available.

It is anticipated that MPFT staff will discuss these opportunities with their line manager, including their research skill needs and desired level of engagement. Department leads will also be expected to consider the skill mix within their teams to discern the value of engagement and level of need.

The following video gives an introduction to the STARS programme and may help to answer any questions you have. We also have some FAQs on our intranet page under the Q&As section. For any other queries, please contact us on STARS@mpft.nhs.uk.

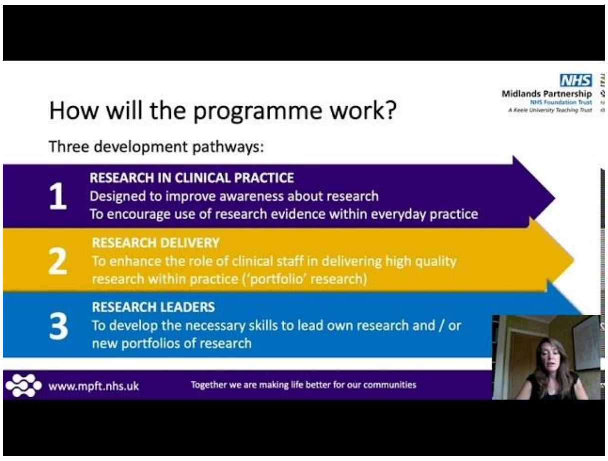

2 - Introduction to STARS by Professor Eleanor Bradley

Pathway 1: Research in Clinical Practice

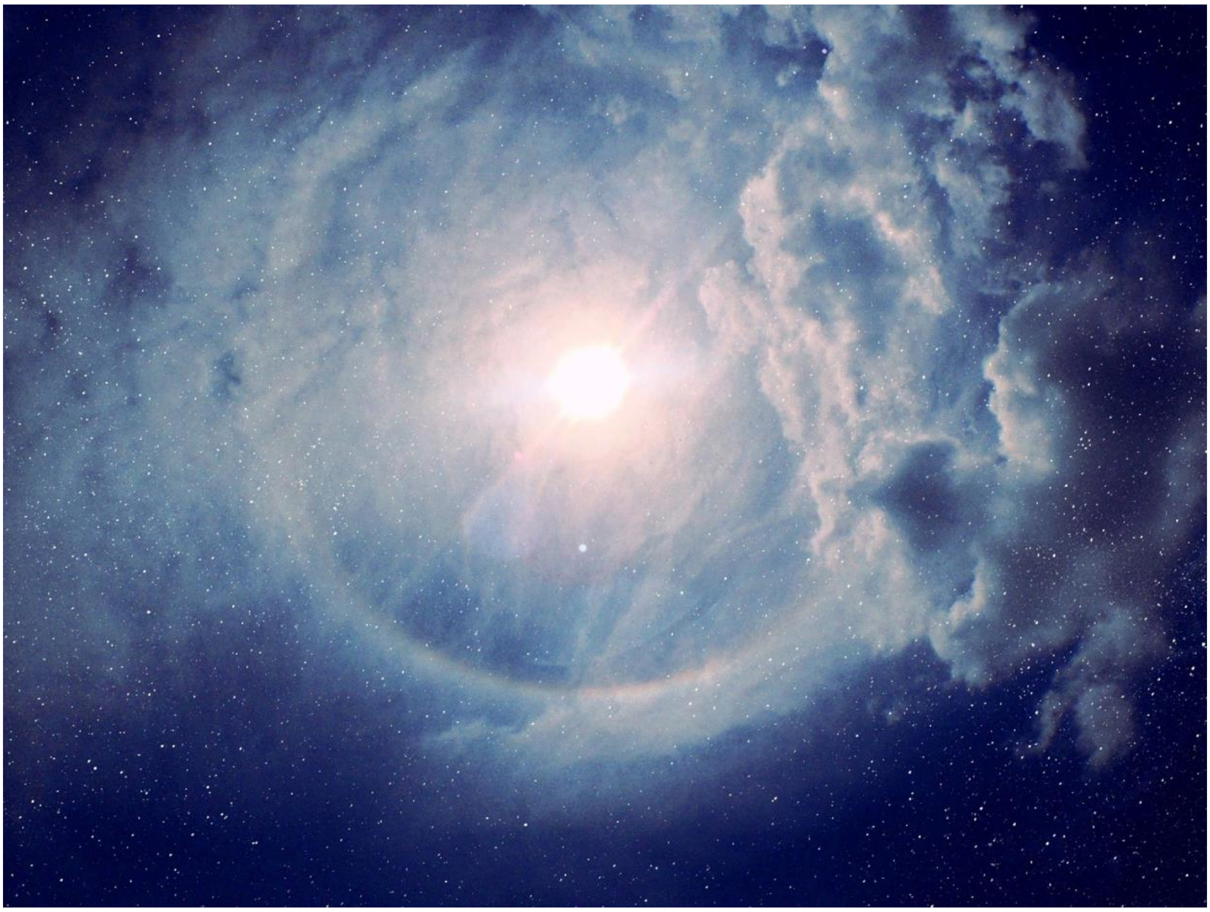

Pathway leads

Kay Stevenson, Senior Knowledge Mobilisation Fellow and Honorary Professor Musculoskeletal Care and Leadership

Lucy Huckfield, Spinal Extended Scope Physiotherapist and Research Facilitator

Overview

Welcome to the Research in Clinical Practice Pathway*. During these workshops we hope to share some practical tips and skills about how research can be useful in your daily working life. It will be suitable for clinicians and managers. We will be using real-life examples, our collective experience of evaluating practice and some tried and tested methods that have been used within MPFT to assist clinicians and leaders to find, evaluate and use evidence in their daily work.*

All sessions will be delivered on MS Teams and will be approximately 3-hours long. Presentation aspects of workshops will be recorded. Session leaders will be available 15 minutes prior to the start of the session to meet and greet learners. Preparatory work may be needed for some sessions.

Please book a place by completing a *booking form* and email to *STARS@mpft.nhs.uk*.

Please note we can only accept bookings up to a week before sessions to give time for any preparatory work.

Workshops within this pathway:

Workshops will be delivered by topic-specialists and will focus specifically on developing skills in evidence identification, interpretation, appraisal and implementation. Learners will also have the opportunity to learn practical skills in audit and evaluation. Each session has been designed to include a mix of taught and practical elements to support interaction and engagement with the training materials.

CRITICAL APPRAISAL SKILLS: QUANTITATIVE RESEARCH

20 January 2021

9.00am - 12.00pm

Session Leads: Fiona Rees, Librarian and Lucy Huckfield, Spinal Extended Scope Physiotherapist and Research Facilitator

Audience:

Most suitable for those in clinical roles, but principles applicable in leadership or management roles

By the end of this session you should:

- Understand what critical appraisal is
- Know what appraisal tools to use and where to find them
- Understand how to develop a clinical question
- Understand what quantitative research is
- Have undertaken critical appraisal of a quantitative research article in a group session

CRITICAL APPRAISAL SKILLS: QUALITATIVE RESEARCH

26 January 2021

9.00am - 12.00pm

Session Leads: Dr Tom Kingstone, Research Associate in Mental Health & Lucy Huckfield, Spinal Extended Scope Physiotherapist and Research Facilitator

Audience:

Most suitable for those in clinical roles, but principles applicable in leadership or management roles

By the end of this session you should:

- Understand what critical appraisal is
- Know what appraisal tools to use and where to find them
- Understand the general purpose of qualitative research and basic designs and methods
- Have undertaken critical appraisal of a qualitative article in a group session

DOING A RAPID LITERATURE REVIEW

23 February 2021

9.00am - 12.00pm

Session Leads: Professor Saeed Farooq, Professor of Psychiatry and Public Mental Health at Keele University, Fiona Rees, Librarian and Lucy Huckfield, Spinal Extended Scope Physiotherapist and Research Facilitator

Audience:

Most suitable for those in clinical roles

By the end of this session you should be able to:

- Know how to make an answerable clinical question to ensure a successful search
- Know where to look to see if your clinical question has already been answered
- Formulate a search strategy for a clinical database search
- Tailor your search to find the best evidence to answer your question
- Organise your results to draw conclusions and make decisions on how it affects your practice

INTERPRETING RESEARCH FINDINGS

17 March 2021

9.00am - 12.00pm

Session Leads: Dr Kay Stevenson, Senior Knowledge Mobilisation Fellow and Honorary Professor Musculoskeletal Care and Leadership and Lucy Huckfield, Spinal Extended Scope Physiotherapist and Research Facilitator

Audience:

Suitable for those in clinical, non-clinical, leadership and management roles

By the end of this session you should have an idea of:

- Why we have different types of research
- What sort information we can draw out of research to help us in our different roles
- Where can we find help to assist us in assessing the quality of a research evidence
- What it feels like to use a quality assessment tool to evaluate the quality of a research study

IMPLEMENTING RESEARCH INTO YOUR WORK

14 April 2021

9.00am - 12.15pm

Session Leads: Dr Kay Stevenson, Senior Knowledge Mobilisation Fellow and Honorary Professor Musculoskeletal Care and Leadership and Lucy Huckfield, Spinal Extended Scope Physiotherapist and Research Facilitator

Audience:

Suitable for those in clinical, non-clinical, leadership and management roles

By the end of this session you should have an understanding of:

- The types of research evidence and its usefulness in your role
- A method that can engage your team in improving the quality of your services and care (Critically Appraised Topic-CAT)
- The opportunities created by a Critically Appraised Group

SERVICE EVALUATION AND AUDIT

13 May 2021

9.00am - 12.15pm

Session Leads: Dr David Dobel-Ober, Evaluation Lead and Steven Hazeldine, Clinical Audit Team Leader

Audience:

Suitable for those in clinical, non-clinical, leadership and management roles

By the end of this session you should have a broad understanding of:

- Understand the definitions of clinical audit & service evaluation in an NHS context
- Clinical audit & service evaluation governance in the Trust
- Key principles of best practice for clinical audit
- The most common approaches to service evaluation

Pathway 2: Research Delivery

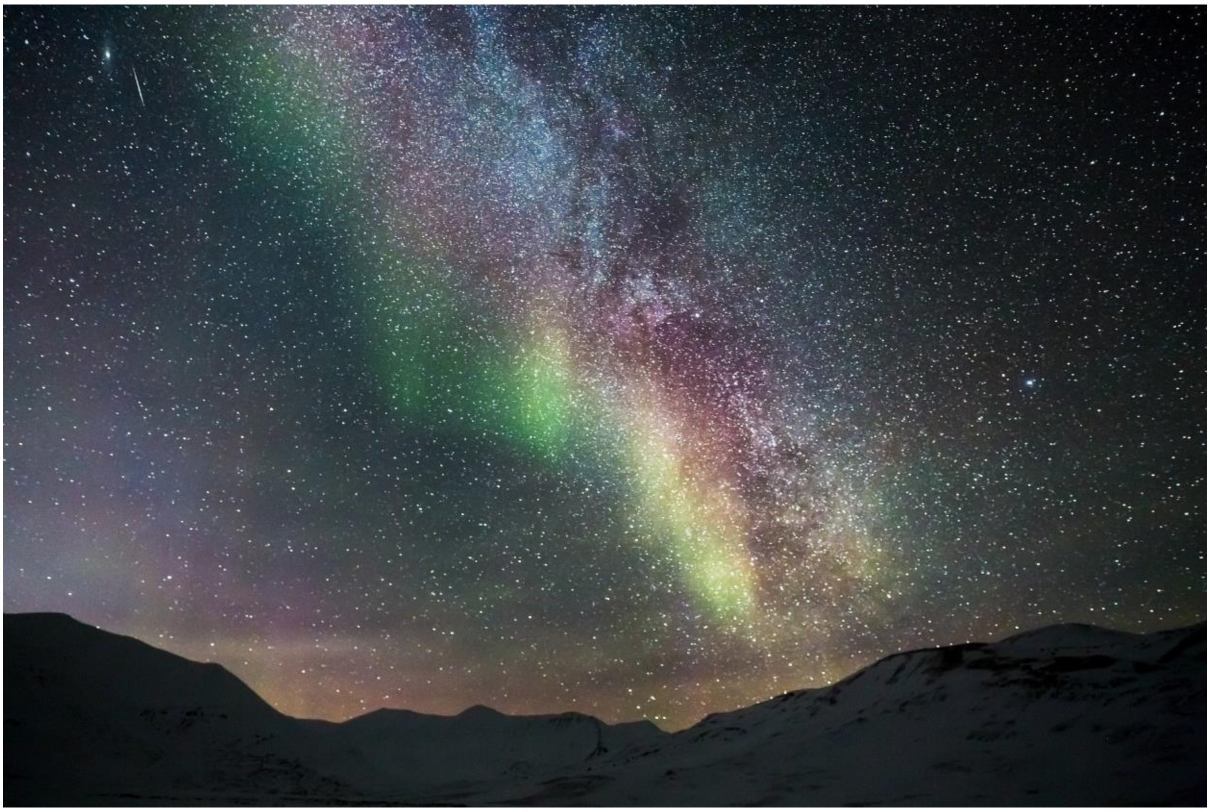

Pathway leads

Liz Glaves, Research Delivery Manager

Paula Coventry, Research Nurse

Overview

Welcome to the Research Delivery Pathway*. This pathway aims to equip staff with the knowledge and skills necessary to take an active role in delivering research led by internal or external teams. Workshops are based on approved training created by the National Institute for Health Research: Clinical Research Network (NIHR CRN). Workshops will focus specifically on topics including introduction to trials, research governance, study processes and procedures, and data quality and monitoring.*

All sessions will be delivered either face-to-face and/or via MS Teams and will be up to 3 hours long. Presentation aspects of workshops will be recorded. Session leaders will be available 15 minutes prior to start of the session to meet and greet learners. Preparatory work may be needed for some sessions.

Please note, the workshops for this Delivery pathway are currently streamlined due to other commitments to COVID-19 research. We hope to offer more training sessions as the year progresses.

Please book a place by completing a _booking form_ and email to _STARS@mpft.nhs.uk_.

Please note we can only accept bookings up to a week before sessions to give time for any preparatory work.

Workshops within this pathway include:

SETTING UP A STUDY: GOVERNANCE PROCESSES

24 February 2021

10.00am - 12.00pm

Session Leads: Susan Lavender, Clinical Research Practitioner and Chantel-Lea Grocott, Research Support Team Leader

Audience:

Anyone with an interest in research

By the end of this session learners should be able to:

- Gain knowledge of the research study processes involved to prepare for the delivery phase.
- To gain awareness of the Governance Processes and regulatory requirements involved to progress the study to the delivery phase

INFORMED CONSENT

27 April 2021

10.00am - 11.30am

Session Leads: Paula Coventry, Research Nurse and Kim Thompson, Research Team Leader

Audience:

All Clinicians who may potentially be involved in approaching service users with study information and consenting into a study.

By the end of this session learners should be able to:

- Understand the ethical and legislative frameworks that underpin, the research consent process
- Understand the principles and responsibilities of consent in clinical research
- Develop an understanding of the research consent process
- Demonstrate high quality practices to support the process
- Gain an insight into the added protection required for vulnerable groups
- Build confidence in participating in the research consent

SITE FILE MANAGEMENT

17th *June 2021*

10.00am

Session Leads: Tim Lewington, Clinical Research Practitioner and Leigh Franks, Clinical Research Practitioner

Audience:

Clinicians who will be tasked to maintain essential documents for a study

By the end of this session learners should be able to:

- Understand the purpose of an Investigator Site File (ISF)
- Identify the essential documents required in an ISF
- Understand the importance of maintaining an ISF
- Set up and maintain an ISF

DATA MANAGEMENT & CRF DESIGN, DEVELOPMENT & COMPLETION

(Formerly Data Quality and Case Report Form Completion)

9 *September 2021*

10.00am - 11.30am

Session Leads: Paula Coventry, Research Nurse and Lucy Hamilton, Research Nurse

Audience:

Clinicians involved in obtaining data in any form for studies

By the end of this session learners should be able to:

- Understand the significance of data management and collection
- Understand the importance of CRF design, development and completion
- Understand the role of data management in quality assurance

Pathway 3: Research Leader

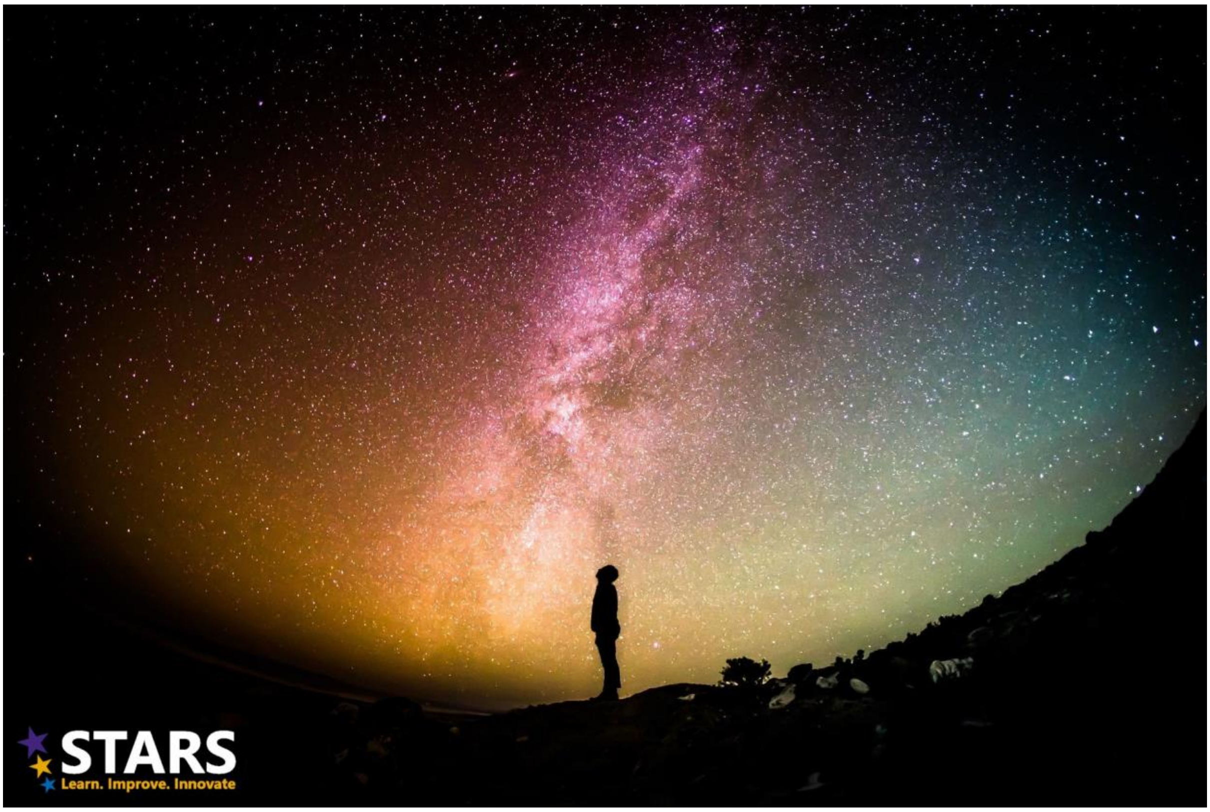

Pathway leads

Professor Eleanor Bradley, Professor of Health Psychology, University of Worcester

Dr Tom Kingstone, Research Associate in Mental Health, MPFT

Overview

Welcome to the Research Leader Pathway. *Workshops in this pathway have been split into introductory and advanced levels to suit existing skills and abilities of intended learners.*

The introductory level workshops aim to equip staff with the knowledge and skills necessary to develop and design their own research project. Introductory workshops are suitable for people new to research or as a helpful refresher.

The advanced level workshops aim to build upon existing knowledge to nurture high level knowledge and skills in research design, analysis and dissemination. Advanced workshops are suitable for people with previous research knowledge and/or experience.

All sessions will be delivered either face-to-face and/or via MS Teams and will be up to 3-hours long. Session leaders will be available 15 minutes prior to start of the session for a meet and greet. Presentation aspects of workshops will be recorded. Preparatory work may be needed for some sessions.

Please book a place by completing a *booking form* **and email to** *STARS@mpft.nhs.uk***.**

Please note we can only accept bookings up to a week before sessions to give time for any preparatory work.

Introductory workshops in this pathway include:

CREATING A RESEARCH QUESTION

25 February 2021

1.00pm - 4.00pm

Intended learning outcomes include:

- Understand the steps involved with creating a research question
- Develop aims and objectives for their ideas
- Identify key elements of a good research question
- Link their question with aligned research methods and approaches

INTRODUCTION TO SYSTEMATIC REVIEWS

29 March 2021

1.00pm - 4.00pm

Intended learning outcomes include:

- Understand reasons for conducting a systematic review
- Understand systematic review processes and procedures
- Learn about different evidence appraisal tools and techniques
- Learn about different approaches to data synthesis

INTRODUCTION TO RESEARCH ETHICS: PRINCIPLES AND PROCESSES

5 May 2021

9.00am - 12.00pm

Intended learning outcomes include:

- Understand the importance of ethics in health and social care research
- Understand key ethical principles and guidelines
- Learn how to apply situational ethics and ethics in process
- Learn about the role of research ethics committees and applying for approvals

INTRODUCTION TO PHILOSOPHY OF RESEARCH

21 June 2021

9.00am - 12.00pm

Intended learning outcomes include:

- Consider contemporary debates as related to the nature of research and research philosophy
- Utilise key terms in relation to research philosophy
- Understand why an understanding of research philosophy is important during early stage of research design
- Critique the meaning of knowledge across different disciplines
- Develop a systematic process of enquiry for new research studies

INTRODUCTION TO DATA COLLECTION (QUANTITATIVE AND QUALITATIVE)

22 July 2021

1.00pm - 4.30pm

Intended learning outcomes include:

- Understand different types of qualitative data and methods of data capture (including novel methods)
- Appreciate the ethical considerations associated with qualitative data collection
- Make a detailed plan for data collection, including when to stop collecting data

INTRODUCTION TO DATA ANALYSIS (QUANTITATIVE AND QUALITATIVE)

6 September 2021

1.00pm - 4.30pm

Intended learning outcomes include:

- Choose between different types of analysis
- Decide whether to use analysis software
- Identify key analysis steps and procedures
- Understand how to present a completed analysis

Advanced workshops in this pathway include:

Please note, dates for this pathway will be announced in Spring 2021. As soon as they are confirmed, we will email all staff who registered an interest in the STARS programme - if you would like to be emailed but are not sure if you are on our list, please get in touch at STARS@mpft.nhs.uk**.**

ADVANCED QUANTITATIVE DESIGN AND ANALYSIS

TBC September 2021

Details coming soon

ADVANCED QUALITATIVE DESIGN AND ANALYSIS

TBC October 2021

Details coming soon

MIXED METHODS RESEARCH

TBC November 2021

Details coming soon

META-ANALYSIS

TBC December 2021

Details coming soon

QUALITATIVE SYNTHESIS

TBC January 2022

Intended learning outcomes include:

- Understand the purpose of a qualitative review synthesis
- Differentiate between different types of systematic review
- Consider why and how to approach a qualitative synthesis
- Outline challenges associated with the approach
- Understand practical techniques to use when conducting a meta-synthesis

INNOVATIVE DISSEMINATION METHODS

TBC February 2022

Intended learning outcomes include:

- Be aware of who your interested stakeholders are
- Have an introduction to the Community of Practice Methodology

STARS Seminar Series

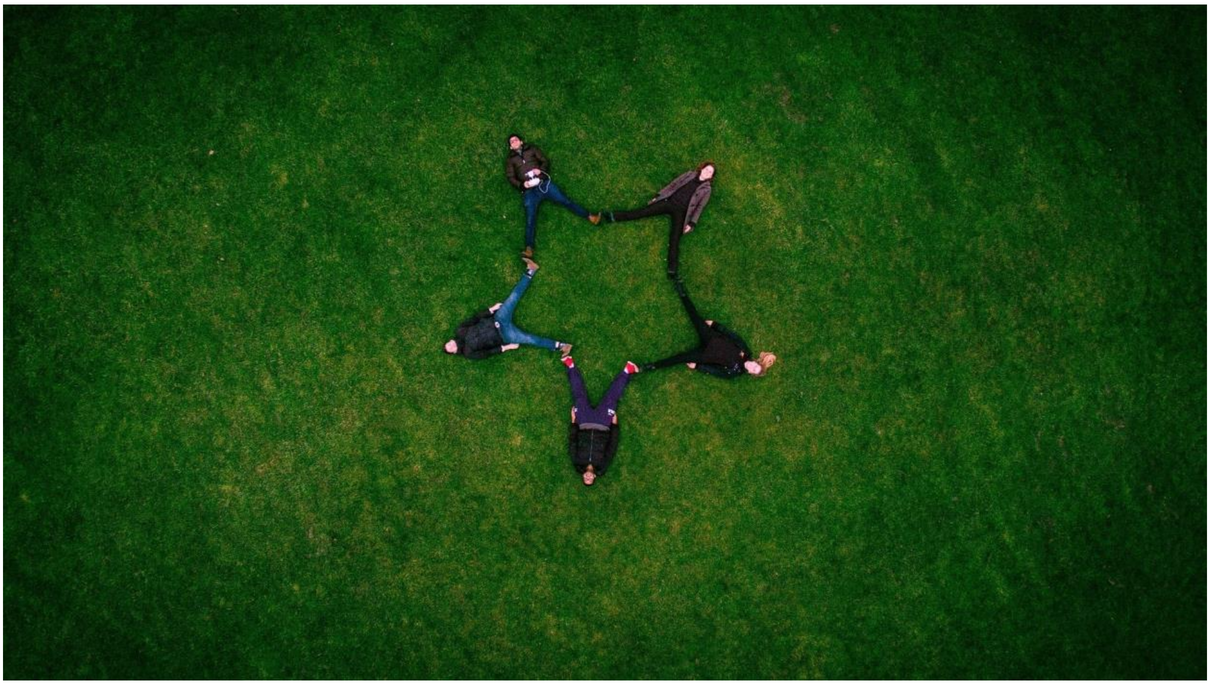

To supplement the dedicated pathways a range of seminars are also planned. Seminars will aim to deliver bite-size learning opportunities to support staff to develop specific research competencies.

The seminars are designed to be flexible to meet the needs of staff looking to obtain specific skills – in the long-term the content will be made available online, for example using YouTube or MS Teams.

Each seminar is designed to be brief, lasting for between 30-45 minutes; dedicated time will be given at the end of each seminar to provide one-to-one support for staff with specific queries.

All sessions will be delivered on MS Teams. Preparatory work may be needed for some sessions.

The seminar list will continue to evolve and expand. Further updates will be shared via social media and will be highlighted on the dedicated website.

Please book a place by completing a booking form **and email to** STARS@mpft.nhs.uk**.**

HOW TO ... DEVELOP A RESEARCH QUESTION

19 January 2021

11.00am - 12.00pm

Session Lead: Professor Saeed Farooq

By the end of this session attendees will:

- Understand the steps involved with creating a research question
- Develop aims and objectives for their ideas
- Identify key elements of a good research question

HOW TO … BALANCE YOUR CLINICAL AND ACADEMIC COMMITMENTS

11 February 2021

11.00am - 12.00pm

Session Leads: Professor Saeed Farooq & Professor Kay Stevenson

By the end of this session attendees will:

- Have top tips on time management
- Understand the benefits of linking academic and clinical priorities

HOW TO … IDENTIFY FUNDING OPPORTUNITIES

9 March 2021

12.00pm - 1.00pm

Session Leads: Dr Tom Kingstone and Gulshan Tajuria

By the end of this session attendees will:

- Understand how to search for and identify relevant funding opportunities
- Review example guidelines and eligibility criteria for submission
- Understand what makes a successful funding application

HOW TO … WRITE AN IRAS APPLICATION

20 April 2021

12.00pm - 1.00pm

Session Leads: Dr Tom Kingstone and Frances Davies

By the end of this session attendees will:

- Understand what the Integrated Application System (IRAS) is and what to expect from the submission process
- Understand how to prepare yourself to complete an application in IRAS
- Learn useful tips on how to complete each section of the application form

HOW TO … INVOLVE PATIENTS AND THE PUBLIC IN RESEARCH

6 May 2021

3.00pm - 4.00pm

Session Leads: Jessica Tunmore and Dr Tim Lewington

By the end of this session attendees will:

- Understand what patient and public involvement in research means
- Distinguish between involvement and engagement in research
- Review examples of PPIE in research undertaken at MPFT
- Identify PPIE resources and sources of support

HOW TO … GET YOUR RESEARCH PUBLISHED

8 June 2021

11.00am - 12.00pm

Session Leads: Professor Eleanor Bradley and Dr Paul Campbell

By the end of this session attendees will:

- Have familiarity with academic writing
- Be able to create the right narrative
- Have skills for creating a clear argument
- Be able to know how to pick the right journal
- Be familiar with the processes of submission
- Be able to respond to reviewer comments
- Have strategies to create impact
- Know what to consider when writing for particular audiences
- Learn how to pitch your article: writing a convincing abstract and lay summary

HOW TO … APPLY FOR ETHICAL APPROVAL

5 July 2021

12.00pm - 1.00pm

Session Leads: Frances Davies and Dr Tom Kingstone

By the end of this session attendees will:

- Have learned about the role of research ethics committees
- Understand how and when to apply for ethical approval
- Have learned some useful tips on completing an ethics application form (including examples)
- Identify useful ethics guidance and resources

HOW TO WRITE A FUNDING APPLICATION

18 August 2021

11.00am - 12.00pm

Session Leads: Professor Saeed Farooq and Professor Athula Sumathipala

By the end of this session attendees will:

- Understand the different funding streams and how to apply using different systems
- Understand how to involve patients and the public in your proposal
- Understand where to seek out advice where required
- Have examples of successful grant applications across MPFT & Keele to understand the key components of a funding application

HOW TO … CONDUCT A LITERATURE REVIEW

16 September 2021

11.00am - 12.00pm

Session Leads: Fiona Rees

By the end of this session attendees will have learned:

- About developing a clear clinical question
- How to use that question to identify the best evidence to answer it effectively

HOW TO … BE A GOOD PRINCIPAL INVESTIGATOR

4 October 2021

12.00pm - 1.00pm

Session Leads: Kim Thompson

By the end of this session attendees will have:

- Practical solutions to empower PIs to achieve effective oversight of their studies
- Had an opportunity for experienced PIs to share their knowledge and experience, as well as an opportunity for those new in the role to gain a greater understanding of their responsibilities and how to achieve them

Additional learning and development opportunities

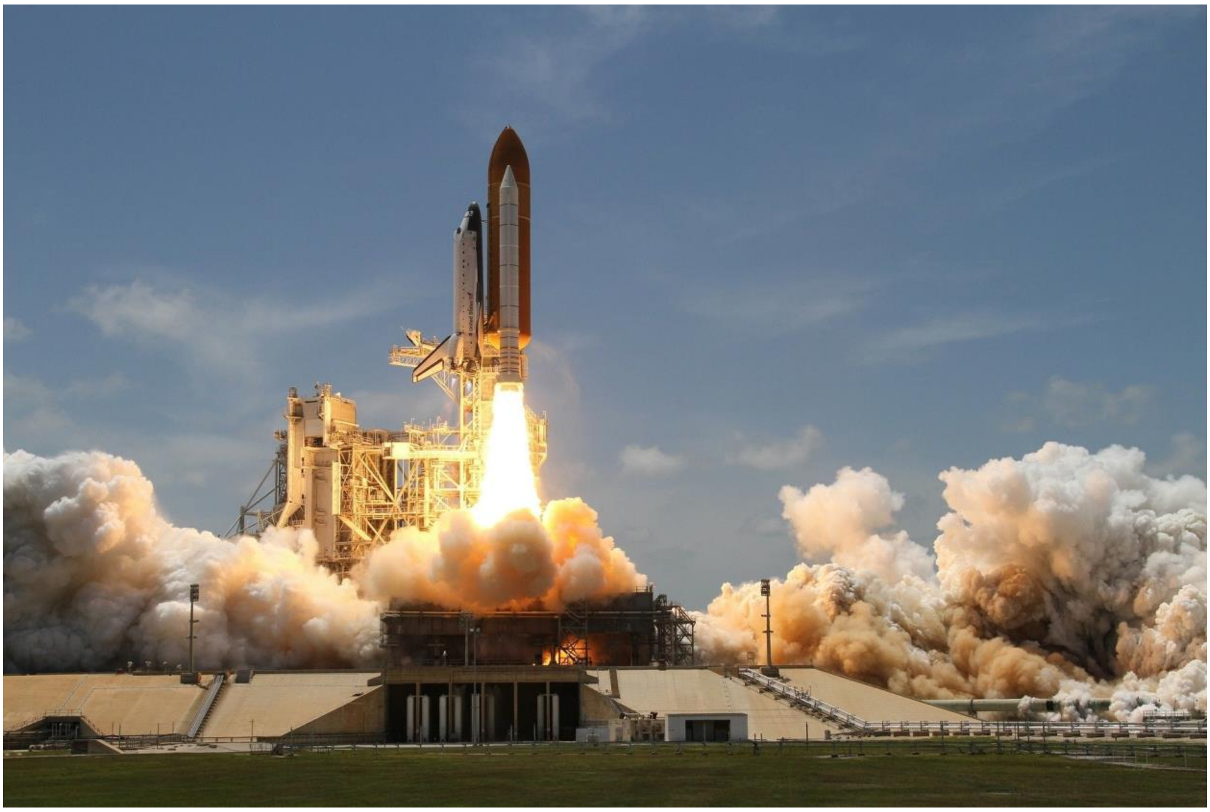

Once you have registered for STARS, we will email you with any updates in relation to the programme, such as new seminars or workshops. You can also keep up-to-date with STARS news and general research learning opportunities in the following ways

- *Follow us on Twitter: @MPFTResearch*
- *STARS intranet page: click here*
- *MPFT Research and Innovation website: click here*

Booking onto learning and development events

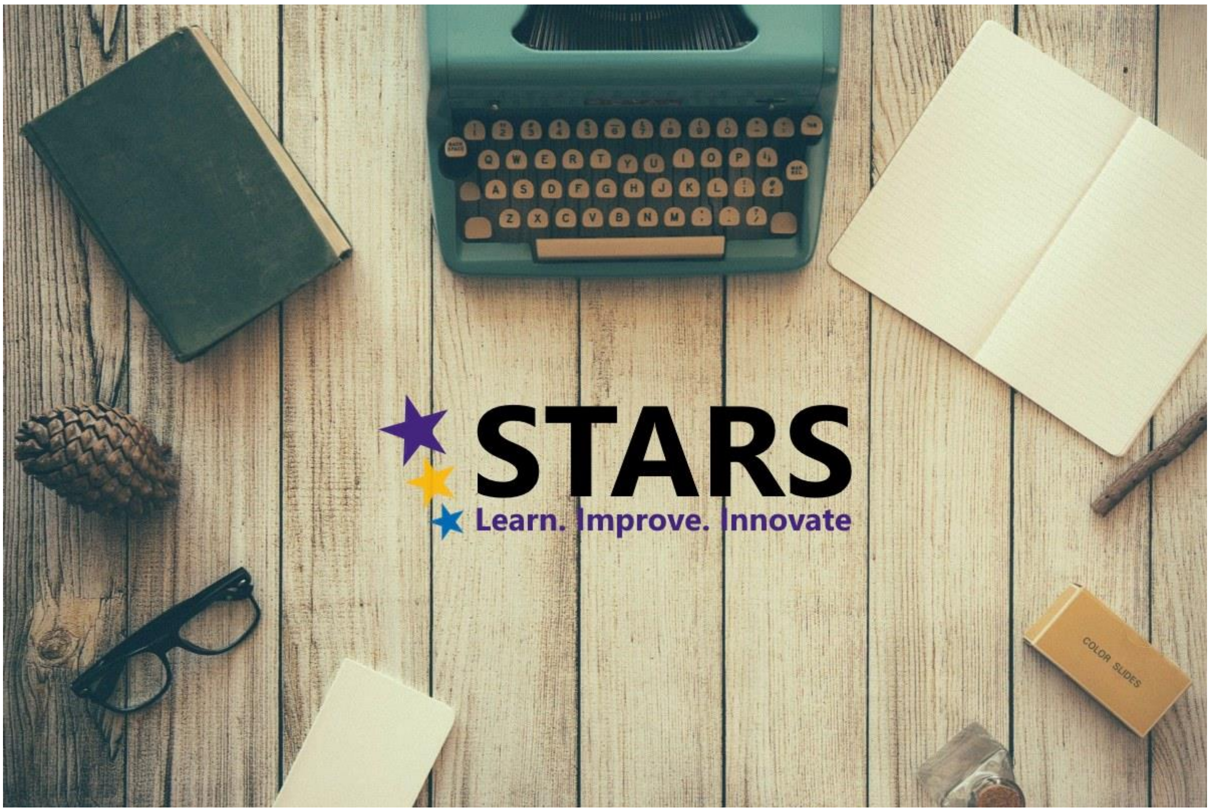

To book a place on any of the workshops or seminars, please complete a booking form and email to STARS@mpft.nhs.uk.

If you have any questions or comments, please don't hesitate to get in touch and email us on STARS@mpft.nhs.uk.

You can also view some Frequently Asked Questions on our intranet page under the Q&A session section here.
