# Supplementary figures and images for: Evaluating the impact of the supporting the advancement of research skills (STARS) programme on research knowledge, engagement and capacity-building in a health and social care organisation in England
Source: BMC Med Educ. 2024 Feb 8;24:126. doi: 10.1186/s12909-024-05059-0 (PMC10854097; doi:10.1186/s12909-024-05059-0)

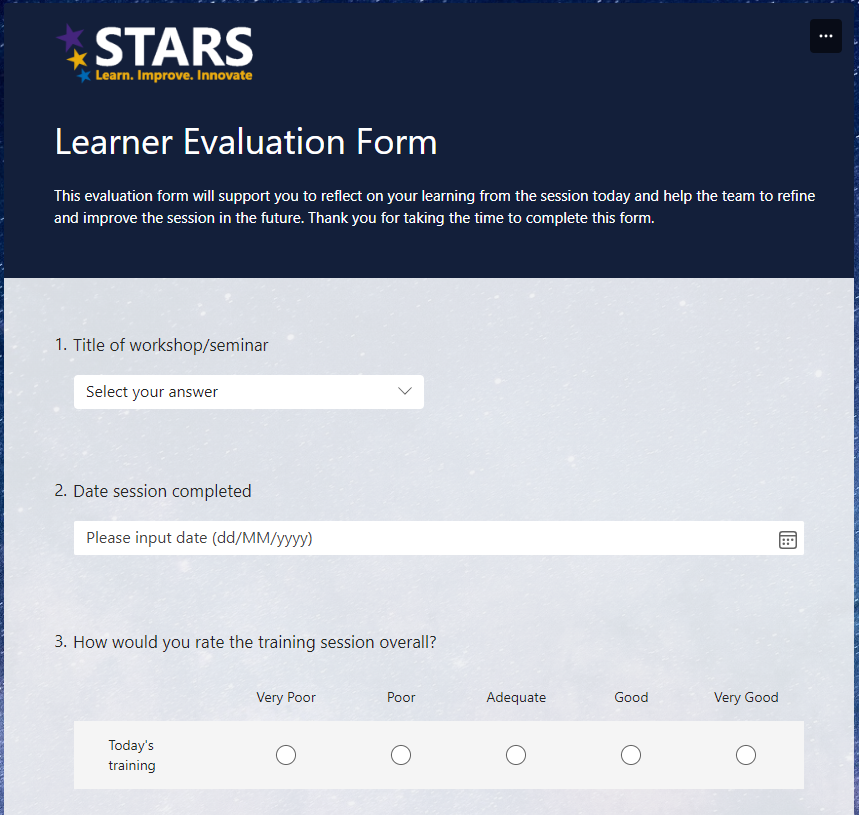

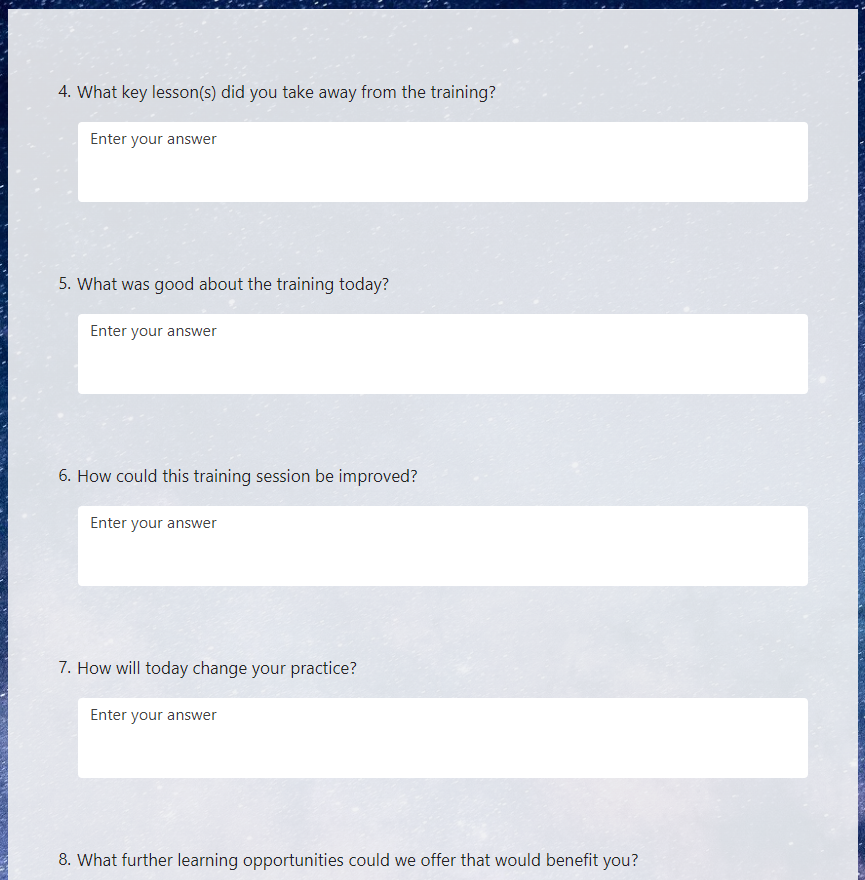


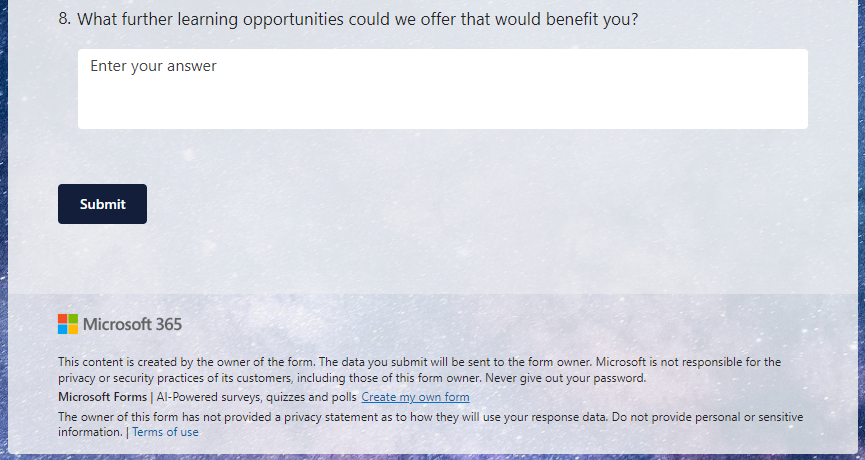

Supplement: Supplementary file 2 — Additional file 2. [file 12909_2024_5059_MOESM2_ESM.docx]
