# Supplementary material for: Evaluating the impact of the supporting the advancement of research skills (STARS) programme on research knowledge, engagement and capacity-building in a health and social care organisation in England
Source: BMC Med Educ. 2024 Feb 8;24:126. doi: 10.1186/s12909-024-05059-0 (PMC10854097; doi:10.1186/s12909-024-05059-0)
Supplement: Supplementary file 3 — Additional file 3. [file 12909_2024_5059_MOESM3_ESM.docx]

**STARS evaluation interview guide**

- Please tell me briefly about your role at MPFT.
- Have you ever been involved in research? (If yes, as a participant or a researcher)
- Purpose or aims- why did you attend the STARS training? (For e.g., personal interest, professional development, additional support for university courses…)
- Please tell something about the overall content of the programme: was there a right balance between the three pathways (research methods, ethics etc and research delivery) and the seminars?
- How did you select which STARS session to attend?
- Based on the sessions attended please comment on what makes an individual session more/less interesting/engaging etc (reflect on the differences between format/presenting style). Views on how the sessions were conducted (content, presenters, reading materials etc).
- What content/session was useful/not so useful? Any specific sessions?
- Use in practice: have you applied the info/knowledge from the sessions into the real world? If so, was it successful (for e.g., if attended qualitative methods session and carried out qualitative project – did it help?)
- Regarding the key barriers to support staff engagement with STARS is there any training/s that you have not been able to attend and what were the reasons? Or what are the facilitators that help you to attend STARS and other such trainings?
- Have you tried to access the videos of sessions attended or missed? How was the experience?
- Could the training be delivered any other way? For e.g., the complete pathway over a short period etc.
- Any similar training that you are aware of? Or any suggestions to include new research training?

Thank you for taking part!
